# Three Cases of Primary Central Airway Tumors of Neurological and Neuroendocrine Origin in Children

**DOI:** 10.1016/j.jpedcp.2026.200226

**Published:** 2026-06-30

**Authors:** Patience Fomazou, François Galode, Joris Menard, Hugues Begueret, Jean Delmas, Arnaud Rodriguez, Fanny Morice-Picard, Anne Rullier, Stephanie Bui, Michael J. Fayon

**Affiliations:** 1 Département de Pédiatrie, CIC-P INSERM 1401, CHU Bordeaux, Bordeaux, France; 2 Université de Bordeaux, Centre de Recherche Cardio-thoracique de Bordeaux, INSERM U1045, Bordeaux, France; 3 Département d’Anatomo-Pathologie, CHU Bordeaux, Bordeaux, France; 4 Service de Radiologie pédiatrique, CHU Bordeaux, Bordeaux, France; 5 Service de Chirurgie thoracique, CHU Bordeaux, Bordeaux, France; 6 Service de Dermatologie pédiatrique, CHU Bordeaux, Bordeaux, France

**Keywords:** child, neural crest tumors, surgical procedures, bronchoscopic

## Abstract

Primary bronchopulmonary tumors are rare in childhood and cause symptoms that are often mistaken for asthma, nontypical pneumonia, or chronic bronchitis. From a single tertiary care center, we report 3 cases of such tumors originating from neural crest cells (carcinoid, schwannoma, neurofibroma) and managed by interventional bronchoscopy and/or surgery. Advances in interventional bronchoscopy are essential to ensure optimal care for children with such airway tumors.

## Key Points

• Neural crest-derived tumors are very rare in children.
• They can cause severe intrinsic (endoluminal) or extrinsic obstruction.
• Targeted, minimally invasive interventional bronchoscopy procedures are central to the management of these tumors.

Bronchopulmonary tumors rarely occur before the age of 20 years. Pediatric primary tracheobronchial tumors are heterogeneous and extremely rare, with an overall incidence of 0.0049 per 100 000 children.^1^, ^2^, ^3^ These tumors include both benign lesions (infantile hemangioma, squamous papilloma, inflammatory pseudotumors, leiomyomas, granular cell tumor, juvenile xanthogranuloma, tracheal lipoblastoma, and laryngotracheal chondromas) and malignant lesions (carcinoids, mucoepidermoid carcinomas, rhabdomyosarcoma, leiomyosarcoma, and adenoid cystic carcinoma). The advent of minimally invasive interventional bronchoscopic techniques has revolutionized the management of these tumors, which were traditionally treated with open surgery.^4^ We report the diagnosis, management, and outcomes of 3 cases of primary bronchial and parabronchial tumors of neurological and neuroendocrine origin in children, treated with interventional bronchoscopy (IB) and surgery.

## Case Presentations

### Case 1

Case 1 involves a 15-year-old girl who was born at term with no significant medical history except for atopic dermatitis. At 12 years of age, she presented with chronic wheezing on exertion. Treated with fluticasone/vilanterol, her forced expiratory volume in 1 second (FEV_1_) remained within normal limits, and her growth curves were normal. Her clinical course was marked by recurrent pneumonia in the right lower lobe and once in the left lower lobe (LLL) (Figure 1, A), without bacteriological documentation (5 episodes between October 2021 and August 2024, ages 13-15 years). Immune assays were normal except for low IgG4 levels (0.043 g/L; reference range, 0.110-1.570 g/L). Total IgE was 496 kUI/L, and other laboratory tests, including Interferon-Gamma Release Assays (QuantiFERON), were negative. There was no systemic inflammation. Lung function tests showed the following: percent predicted forced vital capacity (FVC) of 121%, FEV_1_ of 129%, forced midexpiratory flow (FEF_25-75_) of 116%, vital capacity of 120%, FEV_1_/FVC ratio of 0.90, peak expiratory flow of 118%, Raw of 80%, sGaw of 196%, total lung capacity of 129%, functional residual capacity of 144%, and RV of 157%.

**Figure 1:**
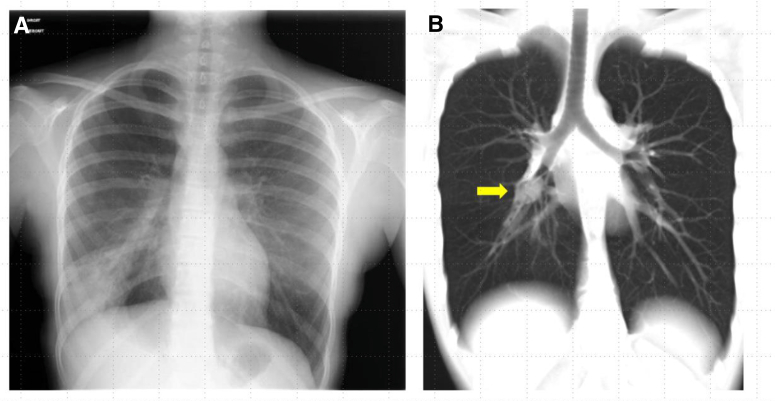
Patient 1. **A,** A chest radiograph shows alveolar consolidation in the right lower lung field and thoracic distension. **B,** An oblique coronal CT scan reconstruction in thick slices averaged to provide an overview of the tracheobronchial tree and lesion (yellow arrow).

At age 13, a chest computed tomography (CT) scan showed airway disease predominantly affecting the RLL, with numerous mucoid impactions and bronchiectasis. There were minimal signs of involvement in the other lobes. Due to recurrent symptoms, flexible bronchoscopy was performed at age 15, revealing a sessile mass completely obstructing one bronchial segment of the right lower lobe (Figure 2, A). A repeat chest CT scan with contrast revealed an endoluminal mass in the right lower lobe (Figure 1, B), associated with mucoid impactions and bilateral grade I bronchiectasis. Her gallium-68 DOTATOC TEP scan was normal. Bronchoscopic biopsy of the lesion revealed medium-sized cells arranged in rows and nests on a richly vascularized thin stroma, giving a neuroendocrine architecture (Figure 3, A and B). The cells were uniform, with regular nucleoli and finely granular chromatin, without mitosis or necrosis. Immunohistochemistry showed expression of chromogranin A and synaptophysin, without thyroid transcription factor-1 expression. This was suggestive of a well-differentiated carcinoid neuroendocrine tumor according to the World Health Organization classification (5th edition),^5^ classified as cT1bN1M0 (TNM 8th classification).^6^ Initial IB using a laser enabled endobronchial debulking of the tumor, followed by right lower and middle lobe surgical lobectomy with radical mediastinal lymph node dissection using robot-assisted surgery. The outcome was favorable, with no tumor recurrence after 2 years. A pictorial representation of the timeline for case 1 is shown Figure 4 (upper panel).

**Figure 2:**
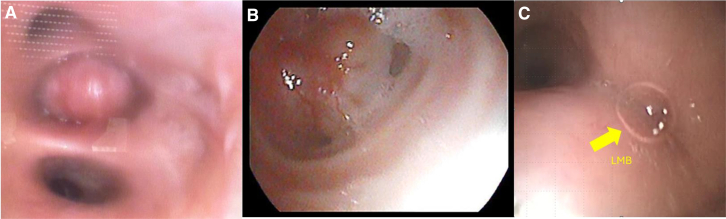
**A,** Patient 1. Complete obstruction of a segment of the right lower lobe by a sessile mass. **B,** Patient 2. Presence of a large tracheal obstructive lesion. **C,** Patient 3. Extrinsic compression of the left main stem bronchus (LMB).

**Figure 3:**
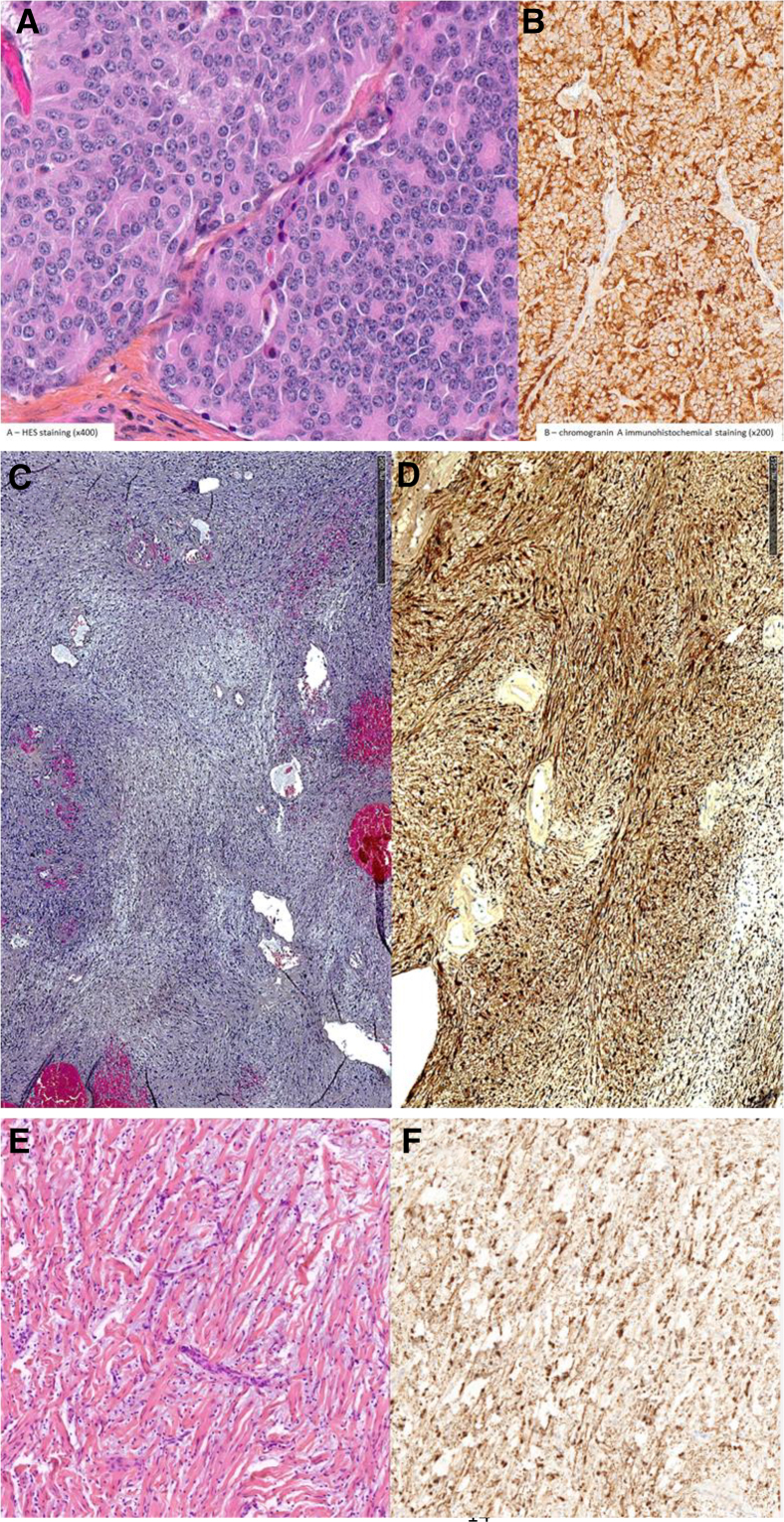
Patient 1: **A,** well-differentiated neuroendocrine tumor tissue without mitosis or necrosis (hematoxylin and eosin); **B,** diffuse cytoplasmic chromogranin A expression. Patient 2: **C,** proliferation of undulating spindle cells (hematoxylin and eosin); immunohistochemistry (**D**): diffuse expression of S100 protein consistent with a submucosal schwannoma. Patient 3: **E,** tumor proliferation combining spindle cells with dense fibro-collagenous tissue (hematoxylin and eosin, ×10); **F,** expression of protein S100 on tumor cells (immunostaining, ×10). **A:** HES staining, magnification ×400 **B:** Chromogranine A immunostaining, magnification ×200 **C:** HES staining, magnification ×50 **D:** Protein S100 immunostaining, magnification ×50 **E:** HES staining, magnification ×100 **F:** Protein S100 immunostaining, magnification ×100

**Figure 4:**
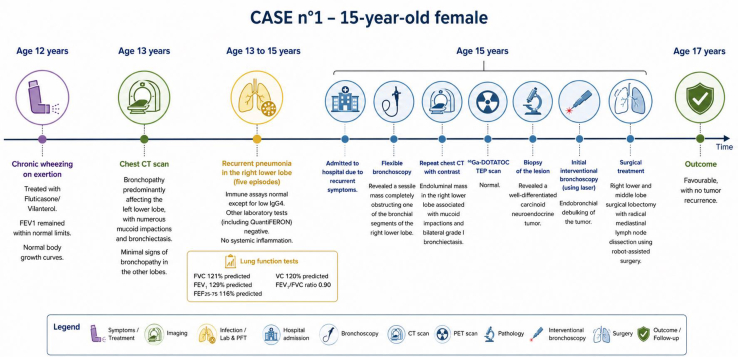

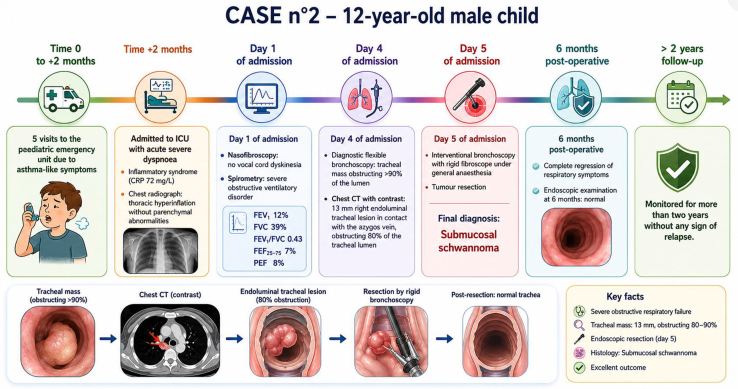

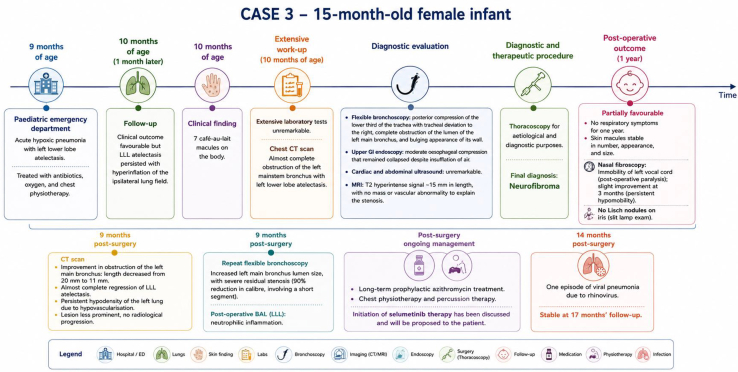
Chronological timelines for the 3 cases presented.

### Case 2

A 12-year-old boy who did not speak French made 5 visits to the pediatric emergency unit over 2 months for asthma-like symptoms associated with anxiety, dry cough, and variable digestive symptoms (nausea, dysphagia). His height and body weight *z*-scores were −0.62 and −1.26, respectively. He was admitted to the intensive care unit with acute severe dyspnea with an inspiratory component, without stridor or desaturation, and with a low-grade fever. On admission, his C-reactive protein was 72 mg/L, and the initial chest radiograph showed thoracic hyperinflation without parenchymal abnormalities. On day 1 of admission, nasofibroscopy was normal, not revealing any vocal cord dysfunction. Spirometry showed a severe obstructive ventilatory disorder with a percent-predicted FEV_1_ of 12%, FVC of 39%, FEV_1_/FVC ratio of 0.43, FEF_25-75_ of 7%, and peak expiratory flow of 8%. On day 4 of admission, diagnostic flexible bronchoscopy revealed a tracheal mass obstructing more than 90% of the lumen (Figure 2, B). This lesion was not biopsied at that stage.

A subsequent CT scan of the chest with contrast showed a 13-mm right endoluminal tracheal lesion in contact with the azygos vein, obstructing 80% of the tracheal lumen (Figure 5). Tumor resection was performed by IB using a rigid fibroscope under general anesthesia. This procedure allowed clear visualization of a budding, hypervascular tumor with a sessile base on the right side of the trachea, 1 cm from the carina. Tumor devascularization was initially achieved by laser coagulation at its base and periphery, followed by mechanical debulking with complete extraction of the tumor. Biopsy results (Figure 3, C and D) showed a proliferation of undulating spindle cells, and immunohistochemistry showed diffuse S100 protein expression, consistent with a submucosal schwannoma. Follow-up was favorable, with complete regression of respiratory symptoms, and an endoscopic examination 6 months later was normal. Further lung function tests were not deemed necessary. The child has been monitored for more than 2 years without any sign of relapse. The timeline for case 2 is shown in Figure 4 (middle panel).

**Figure 5:**
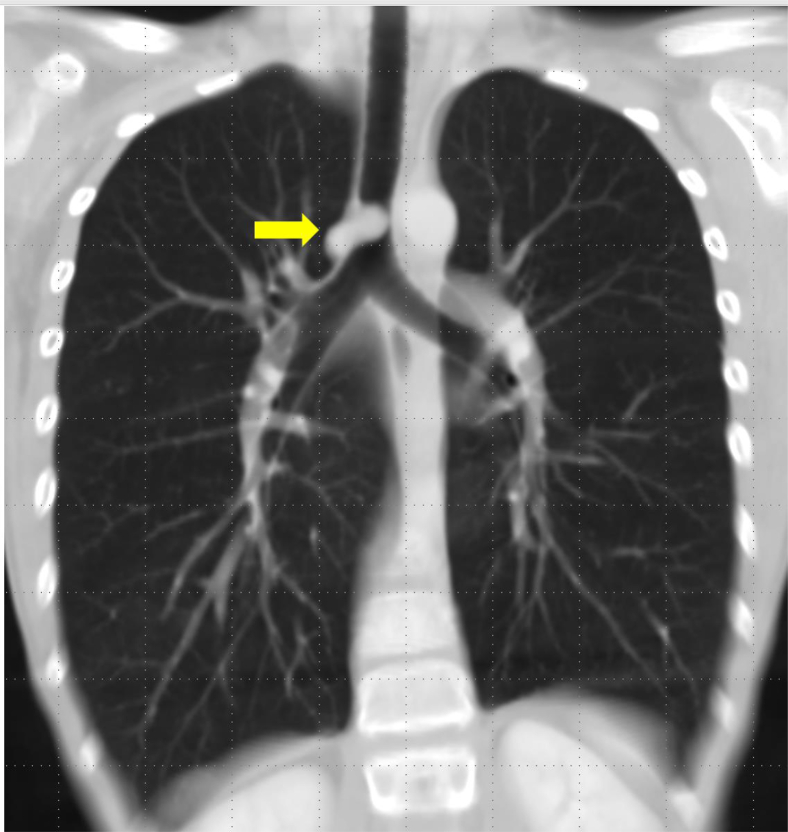
Patient 2. Oblique coronal CT scan reconstructions in thick slices averaged to provide an overview of the tracheobronchial tree and lesion (yellow arrow).

### Case 3

A 15-month-old girl, born at term, was admitted to the pediatric emergency department at 9 months of age with acute hypoxic pneumonia associated with LLL atelectasis. She was treated with antibiotics, oxygen, and chest physiotherapy. One month later, although her clinical outcome was favorable, the LLL atelectasis persisted, along with hyperinflation of the ipsilateral lung field. The child had 7 café-au-lait macules on her body (Figure 6). Extensive laboratory tests were unremarkable.

**Figure 6:**
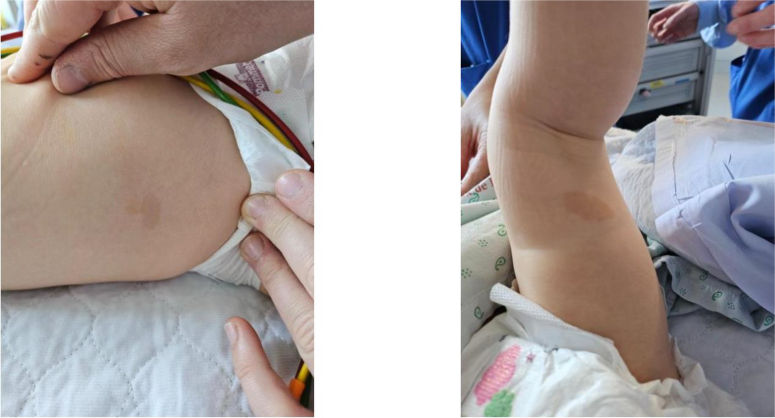
Patient 3. Hyperpigmented macules distributed across the body (7 in total). **Left,** torso. **Right,** thigh.

A CT scan of the chest confirmed almost complete obstruction of the left mainstem bronchus with LLL atelectasis (Figure 7, A). Flexible bronchoscopy revealed posterior compression of the lower one-third of the trachea with tracheal deviation to the right, complete obstruction of the left main bronchus lumen, and a bulging appearance of its wall. Upper gastrointestinal endoscopy showed moderate esophageal compression that remained collapsed despite air insufflation. Cardiac and abdominal ultrasound examinations were unremarkable. Possible extrinsic compression or severe central airway malacia was suspected. Magnetic resonance imaging showed a T2 hyperintense signal approximately 15 mm in length, with no mass or vascular abnormality to explain the stenosis (Figure 7, B).

**Figure 7:**
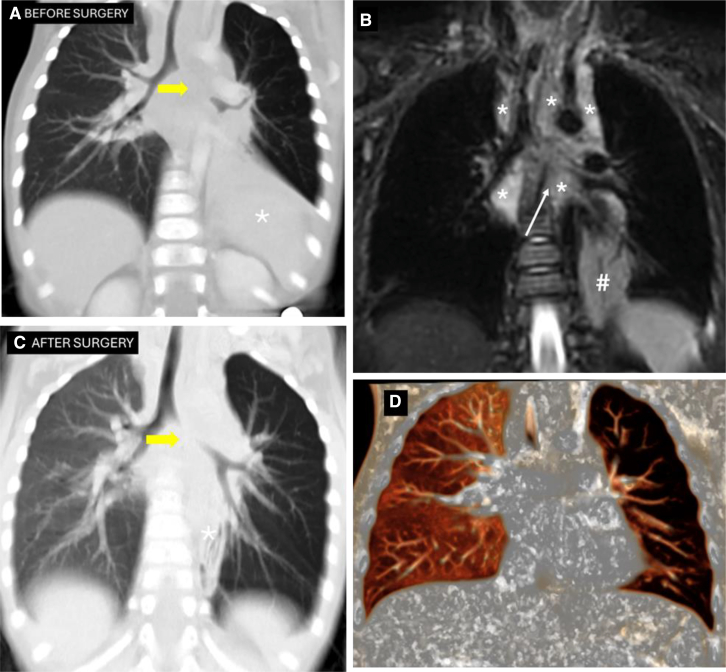
Patient 3. **A, C,** Oblique coronal CT scan reconstructions in thick slices averaged to provide an overview of the tracheobronchial tree and lesion. There is a clear progression between the 2 scans, with less marked stenosis (yellow *arrows*) and resolution of the atelectasis (except for the medial basal segment) (white *asterisks*) after surgery. **B,** T2-weighted slices with fat cancellation (using the Dixon technique) in the 3 planes of space. ∗Plexiform NF. #Atelectasis. The arrow shows the collapsed left main stem bronchus. These images were initially thought to be related to mediastinal lymph nodes and inflammatory changes, but in hindsight they corresponded with the NF1 lesion. **D,** Hyperlucency of the left lobes is mainly explained by hypovascularization rather than distension.

Repeat bronchoscopy with positive pressure insufflation to assess reversibility of the bronchial obstruction showed only partial reversibility, despite positive ventilatory pressures between 30 and 35 cmH_2_O. Insertion of a stent (endoprosthesis) was therefore not considered acceptable, and thoracoscopy for etiological and diagnostic purposes was performed. Elongated plexiform lesions were visualized in the posterior mediastinum, near the pulmonary hilum, on the lateral side of the aorta, and in contact with the main left bronchus (Figure 8, A). Two firm, pearly fragments were extracted (Figure 8, B). Histology of both fragments was virtually identical, showing a proliferation of bland spindle cells with thin, wavy nuclei in a variably loose myxoid or collagenous stroma. These cells were organized in clusters and focally in nodules, giving a plexiform appearance. There was no necrosis or mitotic activity. Immunohistochemistry revealed intense and diffuse co-expression of CD34 and S100 protein, with a low proliferation index (Ki-67 < 1%) (Figure 3, E and F). The final diagnosis was neurofibroma (NF).

**Figure 8:**
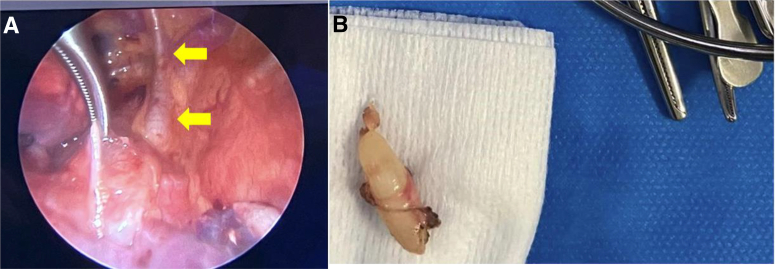
Patient 3. **A,** Elongated plexiform chord-like lesions (yellow arrows) in the left paramediastinal and posterior space. **B,** One of the dissected fragments.

The clinical outcome was partially favorable, with no respiratory symptoms for 1 year. Nasal fibroscopy showed immobility of the left vocal cord (postoperative paralysis), and a follow-up examination 3 months later showed slight improvement (persistent hypomobility). No Lisch nodules were observed on the iris under slit lamp examination. A CT scan performed 9 months later (Figure 7, C) showed improvement in the obstruction of the left main bronchus, which was initially visible over a length of 20 mm, but was now 11 mm long. Almost complete regression of the LLL atelectasis was observed, with persistent hypodensity of the left lung due to hypovascularization. The lesion was difficult to distinguish clearly on imaging, but was certainly less prominent and showed no signs of radiological progression. Repeat flexible bronchoscopy showed increased left main stem bronchus lumen size, although with severe residual stenosis (90% reduction in caliber, involving a short segment). Postoperative bronchoalveolar lavage in the LLL showed neutrophilic inflammation: total cell density, 170 000/mL; differential cell count, macrophages, 36% (reference values, 80%-90%); lymphocytes, 10% (reference value, <20%); neutrophils, 54% (reference value, <10%); and eosinophils, 0% (reference value, <2%). Long-term prophylactic azithromycin, combined with chest physiotherapy and percussion therapy, was prescribed. Considering the symptomatic presentation and lack of surgical options for this internal NF, initiation of selumetinib therapy^7^ has been discussed and will be proposed to the patient’s guardians. The patient experienced 1 episode of viral pneumonia due to rhinovirus 14 months post surgery, but is stable at 17 months’ follow-up. See Figure 4 (lower panel) for the timeline.

This noninterventional study, conducted exclusively using retrospective data from routine clinical care, was carried out in accordance with French regulations on secondary use of health data. Moreover, according to French law and the MR004 regulation, patients received an information form, allowing them to accept or refuse the use of their data for research and publications. The Centre Ethique en Santé de Bordeaux authorized this publication (Authorization number: CER-BDX 2025-396).

## Discussion

Carcinoid tumors, schwannomas, and NFs originate from neural crest cells. When present in the lower respiratory tract, these tumors may cause chronic respiratory symptoms, often mistaken for asthma, atypical pneumonia, or chronic bronchitis. This frequently results in delayed diagnosis, often made at a stage of significant parenchymal destruction or metastasis.

The clinical presentation of these neural crest-derived tumors, as with other tumors in the pediatric population, is nonspecific. Obstructive signs such as stridor, wheezing, or dyspnea have been reported. Most often, children present with chronic cough due to persistent mucosal irritation or ineffective distal drainage. Depending on the degree of luminal obstruction, bronchiectasis and atelectasis may occur, leading to recurrent pneumonia.^8^

Some of these tumors express somatostatin receptors, enabling diagnosis with radioactive somatostatin analogues such as indium-111 pentetreotide (OctreoScan).^8^ In a retrospective study of 16 adults (aged 37-78 years), OctreoScan showed higher sensitivity (77%) than positron emission tomography, which failed to detect 43% of tumors.^8^ Gallium-68 DOTATATE, a positron emission tomography imaging agent used for well-differentiated neuroendocrine tumors, targets somatostatin receptor subtype 2 and provides superior detection compared with indium-111 pentetreotide single photo emission CT with CT due to its approximately 100-fold higher receptor affinity.^9^ Octreotide and DOTATATE imaging apply only to neuroendocrine (carcinoid) tumors and not the others described in this article. Definitive diagnosis requires tissue biopsy obtained by bronchoscopy^10^ or by surgical resection.

Although rare in children, bronchial carcinoid tumors are the most common primary malignant tumors in children (approximately 80% of all malignant tumors) and the most familiar to pediatric pulmonologists.^3^ They arise from neuroendocrine cells in the respiratory epithelium. The typical form has low metastatic potential. The average age of onset is 12 years. The most common radiological finding is a parenchymal mass with lobar or segmental atelectasis. In 75% of cases, it is located in the right bronchial tree, as in our patient.^3^ Carcinoid syndrome is a rare manifestation in children and is most often observed when metastasis is present.^3^

NFs and schwannomas are the 2 main benign tumors of the nerve sheath. Schwannomas in the pediatric population have been reported in the parenchyma or bronchi. Tracheal localization is rare and more frequently found in adults. Malignant degeneration of these tumors is extremely rare.^11^ NFs are most often seen in neurofibromatosis type 1 (NF1), also called Recklinghausen's disease. It is a rare inherited disorder caused by mutations in the NF1 tumor suppressor gene. NF1 is a rare condition in children. NF1 mainly includes cutaneous, ocular, musculoskeletal, neurological, and vascular manifestations, but it can also affect the lungs.^12^ Plexiform NF is the main complication of NF1.^13^ It is present from birth and can grow and develop into a large benign tumor surrounding any nerve in the body, with devastating complications such as obstruction of surrounding structures. There is a 10% risk of malignant degeneration of plexiform NFs, which is responsible for its morbidity and mortality.^14^ The median age at diagnosis of NF1 is 1 year. Medullary, thoracic, and abdominal locations of NF are life threatening and require early surgical intervention before symptoms occur.^11^ Some cases of postoperative relapse (approximately 20%) have been reported, sometimes requiring repeat surgery.^13^

To date, there is no gold standard in pediatrics for the management of bronchial or parabronchial tumors. Radical surgical excision with lobectomy and pneumonectomy have traditionally been performed for decades in the management of bronchial and parabronchial tumors in children. However, methods that preserve the remaining lung parenchyma, especially if it is healthy, are now recommended.^10^^,^^15^ IB has revolutionized the management of these tumors. It enables conservative resection combined with other specific techniques, such as cryotherapy and laser therapy, thereby limiting long-term complications.^10^ In the present article, IB with flexible and rigid bronchoscopes was used in all 3 cases and completed by surgery. IB is often the first choice for benign pedunculated lesions without any extratracheal extension.^10^^,^^15^ Cryotherapy is becoming increasingly popular for biopsies and is usually performed with a rigid bronchoscope to debulk obstructive endobronchial tumors. The availability of a flexible cryoprobe with a diameter of 1.1 mm, which can pass through the flexible bronchoscope, now allows this technique to be used more frequently in pediatric cases.

Few studies have examined the use of cryotherapy in children. A recent large multicenter US study evaluated the use of cryotherapy and cryobiopsy during pediatric bronchoscopy in 187 patients aged 0-21 years between 2017 and 2025. A total of 317 procedures were performed, most commonly for cryoextraction, cryorecanalization, and cryobiopsy. Results showed high success rates, with cryoextraction and cryorecanalization being at least partially successful in more than 90% of cases, while cryobiopsy achieved successful tissue evaluation in 96% of biopsies and a 73% diagnostic yield. Bleeding was the most frequent complication, but was usually mild, and no cases of pneumothorax were reported, indicating that these procedures are generally safe and effective when performed at specialized pediatric centers.^4^ This method is advantageous because the risk of bleeding or stenotic scarring is reduced, but it requires a well-trained and experienced team. Other methods, such as laser resection with or without CO_2_, microdebridement, and electrocautery, can also be used, but with considerable adverse effects.^10^^,^^15^

The 3 cases of neural crest-derived tumors described in our study all had favorable outcomes because further investigations were carried out early, when a differential diagnosis of allergic asthma or a respiratory condition unresponsive to initial treatment was suspected. Although rare, every pediatrician or pulmonologist should consider a differential diagnosis in cases that fail to respond to standard therapies (Figure 9). The IB and surgical methods used to treat our cases have proven effective, with no recurrences of the illness to date.

**Figure 9:**
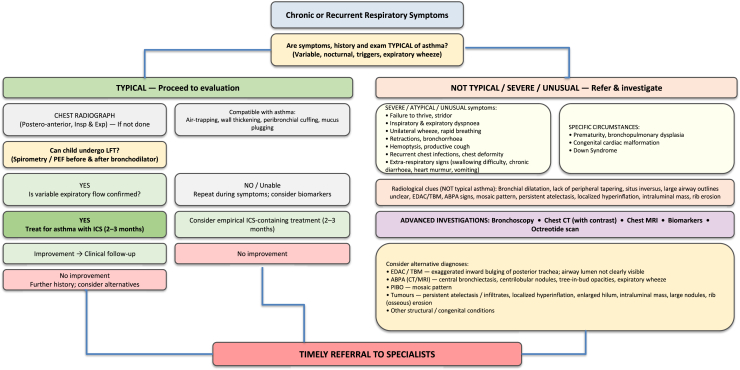
Pediatric Chronic / Recurrent Respiratory Symptoms — Diagnostic Algorithm.

In conclusion, it is essential to improve the diagnostic and therapeutic methods for pediatric airway tumors. The management of tumors in children has been transformed by more conservative treatment approaches, which have improved the long-term prognosis. Targeted minimally invasive IB procedures, such as laser, argon plasma coagulation, and cryotherapy, can be used in conjunction with surgical management for neuroendocrine tumors.

## CRediT authorship contribution statement

**Patience Fomazou:** Writing – original draft. **François Galode:** Validation, Investigation. **Joris Menard:** Validation, Investigation. **Hugues Begueret:** Resources. **Jean Delmas:** Resources. **Arnaud Rodriguez:** Resources. **Fanny Morice-Picard:** Resources. **Anne Rullier:** Resources. **Stephanie Bui:** Writing – review & editing, Visualization, Investigation. **Michael J. Fayon:** Writing – review & editing, Validation, Supervision, Conceptualization.

## Declaration of Competing Interest

We have no competing interests to declare, and confirm that there are no relevant financial or nonfinancial competing interests to report.
